# Clinically meaningful and lasting HbA_1c_ improvement rarely occurs after 5 years of type 1 diabetes: an argument for early, targeted and aggressive intervention following diagnosis

**DOI:** 10.1007/s00125-018-4574-6

**Published:** 2018-02-24

**Authors:** Krishnarajah Nirantharakumar, Nuredin Mohammed, Konstantinos A. Toulis, G. Neil Thomas, Parth Narendran

**Affiliations:** 1 Department of Diabetes, University Hospitals Birmingham NHS Foundation Trust, Birmingham, UK; 2 Public Health, Epidemiology and Biostatistics, Institute of Applied Health Research, College of Medical and Dental Sciences, University of Birmingham, Edgbaston, Birmingham, B15 2TT UK; 3 Institute of Immunology and Immunotherapy, Research College of Medical and Dental Sciences, University of Birmingham, Edgbaston, Birmingham, B15 2TT UK

**Keywords:** Glycated haemoglobin, HbA_1c_, Tracking, Type 1 diabetes mellitus

## Abstract

**Aims/hypothesis:**

Our objectives were to explore whether the phenomenon of HbA_1c_ ‘tracking’ occurs in individuals with type 1 diabetes, how long after diagnosis does tracking take to stabilise, and whether there is an effect of sex and age at diagnosis on tracking.

**Methods:**

A total of 4525 individuals diagnosed with type 1 diabetes between 1 January 1995 and 1 May 2015 were identified from The Health Improvement Network (THIN) database. Mixed models were applied to assess the variability of HbA_1c_ levels over time with random effects on general practices (primary care units) and individuals within practices.

**Results:**

4525 individuals diagnosed with type 1 diabetes were identified in THIN over the study period. The greatest difference in mean HbA_1c_ measurement (−7.0 [95% CI −8.0, −6.1] mmol/mol [0.6%]) was seen when comparing measurements made immediately after diagnosis (0–1 year since diagnosis) with those at 10 or more years (the reference category). The mean difference in HbA_1c_ for the successive periods compared with 10 or more years after diagnosis declined and was no longer statistically significant after 5 years. In the stratified analysis using sex and age group there was considerable heterogeneity with adult onset type 1 diabetes appearing to track earlier and at a lower mean HbA_1c_.

**Conclusions/interpretation:**

In individuals with type 1 diabetes, glycaemic control measured by HbA_1c_ settles onto a long-term ‘track’ and this occurs on average by 5 years following diagnosis. Age at diagnosis modifies both the rate at which individuals settle into their track and the absolute HbA_1c_ tracking level for the next 10 years.

**Electronic supplementary material:**

The online version of this article (10.1007/s00125-018-4574-6) contains peer-reviewed but unedited supplementary material, which is available to authorised users.

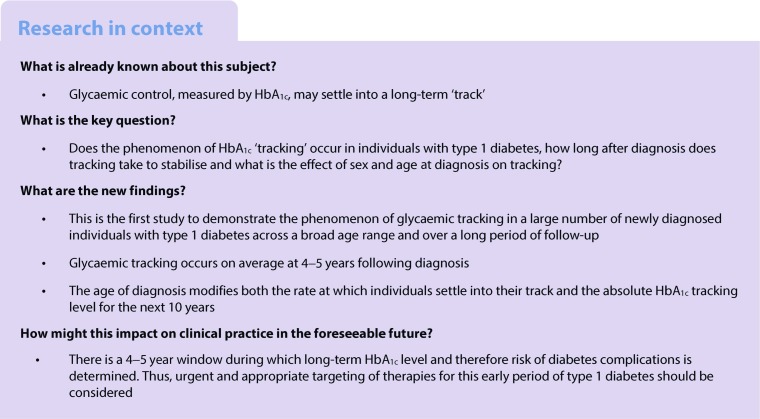



## Introduction

Clinical practice suggests that HbA_1c_ remains remarkably stable in individuals with type 1 diabetes. Some individuals are consistently able to achieve good glycaemic control at repeated clinical follow-up, while others struggle to do so for any meaningful period of time. In these latter individuals, life events (such as pregnancy) or planned interventions (including structured education or continuous subcutaneous insulin infusion therapy) associate with improvements in HbA_1c_. However, this improved HbA_1c_ is often not maintained beyond a few years [1, 2]. This stability of HbA_1c_ that can manifest over decades, or even a lifetime, has been referred to as glycaemic ‘tracking’ [3].

Glycaemic tracking is distinct from HbA_1c_ variability. Year to year variability in HbA_1c_ is a characteristic described in individuals with pre-existing diabetes and associates with both micro- and macrovascular disease [4, 5]. Glycaemic tracking is also not simply the inverse of glucose variability, which is the daily change in blood glucose that includes symptomatic hypo- and hyperglycaemia.

It is vitally important to explore the phenomenon of glycaemic tracking. If tracking is an inherent part of the natural history of type 1 diabetes, this would emphasise the importance of early metabolic control after diagnosis. There may therefore be a window of opportunity where focused interventions set the scene for long-term glycaemic control and facilitate cost effective allocation of time and resources. Exploring the phenomenon of glycaemic tracking may also provide insights into underlying mechanisms, and from there, testable approaches to influencing the final level of HbA_1c_ tracking.

Glycaemic tracking remains to be well characterised. Crucially, previous reports [3–18] have largely examined individuals with pre-existing type 1 diabetes (i.e. not from the time of diagnosis). Those few studies that have followed individuals from the time of diagnosis have been of limited sample size and short duration, have examined individuals across a narrow age group (primarily paediatric) and have not adjusted for important confounders (e.g. socioeconomic status). These studies are summarised in the electronic supplementary material (ESM) Table 1 and illustrate the need to study the phenomenon of glycaemic tracking in a more robust manner.

To explore the concept of glycaemic tracking formally, we performed a large, UK population-based cohort study involving over 4000 individuals with newly diagnosed type 1 diabetes and over 50,000 longitudinally collected HbA_1c_ measurements. We wished to establish: (1) whether the phenomenon of HbA_1c_ tracking occurs in individuals with type 1 diabetes; (2) how long after diagnosis does tracking take to stabilise; and (3) the effect of sex and age of diagnosis at tracking.

## Methods

The Health Improvement Network (THIN) database is a large primary care dataset derived from general practices (primary care units) across the UK [19]. More than 675 practices contribute to the dataset resulting in over 14 million patient records of which around 4 million are active participants. The database consists of individuals’ demographic details, diagnosis, prescriptions and laboratory results. The database is generalisable to the UK population, and has been utilised for numerous epidemiological studies, including type 1 diabetes [20].

The use of the THIN data for research was approved by the South-East Multicenter Research Ethics Committee in 2003, without the need for informed consent. As per the requirements for ethical approval, further registration and authorisation for this project were obtained from the Scientific Review Committee of the data provider (IQVIA: 17THIN015).

### Population

Individuals diagnosed with type 1 diabetes between 1 January 1990 and 1 May 2015 were identified using appropriate Read codes (https://digital.nhs.uk/article/1104/Read-Codes) and based on an algorithm recently published by Sharma et al 2016 [21]. To be classified as having type 1 diabetes, participants need to have a type 1 diabetes specific Read code, be aged less than 40 years at diagnosis and have been prescribed insulin but not oral hypoglycaemic medications. Individuals were eligible to be included in the cohort if they were diagnosed at least 1 year after registration with the practice or a year after the practice was eligible to take part, whichever was the latest. The date of diagnosis used was the one recorded in the database. Participating general practices were eligible to take part following: (1) introduction of the computerised system; (2) the date practices were deemed as having acceptable mortality rates. This was important to ensure that practices were recording important information and comorbidities accurately.

### Measurement of HbA_1c_

All HbA_1c_ values recorded in the database were extracted for the cohort of individuals with type 1 diabetes. Where the unit of measurement was percentage, values were converted to mmol/mol for analysis. Duplicates and implausible measurements (<20 mmol/mol [<4%] and >195 mmol/mol [>20%]) were removed before the analysis. Initial HbA_1c_ at the time of diagnosis is often measured in a secondary care setting and these data were therefore not available for inclusion.

### Statistical analysis

The analysis aimed to answer the three aforementioned questions: does HbA_1c_ tracking occur among individuals with type 1 diabetes; if tracking occurs, how soon after diagnosis does this manifest; if tracking occurs, what is the impact of sex and age at diagnosis on the natural history of tracking?

Sociodemographic characteristics, HbA_1c_ levels and time since diagnosis were summarised using descriptive statistics. Exploratory plots were produced for the mean levels of HbA_1c_ vs time since diagnosis of type 1 diabetes. Similar plots were also produced stratifying by 10 year age bands and sex. The time since diagnosis with type 1 diabetes represents the duration between the date when a particular HbA_1c_ measurement was taken and the initial date of diagnosis. This was divided into 11 categories: 0–1 years, 1–2 years, 2–3 years,…, 9–10 years and ≥10 years to facilitate modelling because our exploratory analyses indicated that the association between HbA_1c_ level and time was likely to be non-linear.

Glycaemic tracking was defined as a period in which there was no statistically significant (*p* < 0.05) difference in HbA_1c_ across adjacent years in comparison with the HbA_1c_ measurement at ≥10 years from diagnosis.

We used linear mixed effects models to assess the variability of HbA_1c_ levels over time with random effects on practices and individuals within practices. These models, also known as multilevel or hierarchical linear models, constitute both fixed effects and random effects. The fixed part is similar to standard linear regression but the addition of random effects allows the model to account for the potential effect of variability at different grouping or clustering levels. In other words, the random effects are variance components associated with each level. In our study, there are three variance components, namely: (1) within individual variability (the residual error associated with repeated measurements from an individual), (2) between individuals within practice variation (as several individuals share the same practice), and (3) between practice variation. Individuals within the same practice may be correlated because of a shared random intercept, through a shared random slope on a covariate or both. Thus, both adjusted and unadjusted two-level random intercepts and slopes (for individuals within practices) models were fitted using the ≥10 years duration group as reference. The adjusted model included age at diagnosis (10 year bands), sex and the Townsend index.

The Townsend index, ranging from 1 to 5, is a measure of material deprivation, calculated using social indices such as income, education and employment specific to each participant’s neighbourhood [22, 23]. Socioeconomic status has been convincingly related to glycaemic control [24] and the Townsend index has been used extensively as a covariate in diabetes studies using THIN database [25–27]. In the adjusted model, the group with the longest duration of type 1 diabetes (≥10 years) was preferred as the reference category; this is because if individuals were to track, i.e., if the proposed hypothesis was true, then we would expect to see no significant difference between this group and groups with a lower duration of type 1 diabetes. The duration after which there is no significant difference in the final HbA_1c_ will be the time point at which tracking manifests. In addition to this, a stratified analysis will be conducted using sex and age group as stratification factors.

In a sensitivity analysis, the random intercepts and slopes model were fitted including data only from individuals with 10 or more complete years of follow-up. All analyses were performed using Stata 14 (StataCorp, College Station, TX, USA).

## Results

There were 4525 individuals diagnosed with type 1 diabetes in the THIN database over the study period 1995–2015 from a total of 617 practices. The majority were male (60.6%) and the highest proportion (38.4%) were diagnosed between the ages of 10 and 20 years. The mean ± SD HbA_1c_ level was 72.6 ± 20.6 mmol/mol (8.8 ± 4%) and the median follow-up after diagnosis was 5 years (Table 1).

**Table 1:** Baseline characteristics of participants and HbA_1c_ summary statistics

Characteristics	All participants *N* = 4525	Participants contributing for 10 years and above *n* = 938
Age group (years)		
0–10	1294 ± 28.6	293 ± 31.3
10–20	1737 ± 38.4	324 ± 34.5
20–30	798 ± 17.6	138 ± 14.7
30–40	696 ± 15.4	183 ± 19.5
Sex		
Male	2743 ± 60.6	574 ± 61.2
Female	1782 ± 39.4	364 ± 38.8
Townsend index		
1	1020 ± 22.5	229 ± 24.4
2	869 ± 19.2	178 ± 19.0
3	923 ± 20.4	190 ± 20.3
4	828 ± 18.3	175 ± 18.7
5	650 ± 14.4	137 ± 14.6
Missing	235 ± 5.2	29 ± 3.0
Follow-up period (years)		
Mean (SD)	6.0 ± 4.6	13.2 ± 2.4
Median (25th–75th percentile)	5.0 (2.1–9.2)	13.2 (11.2–14.8)
HbA_1c_ (mmol/mol)^a^		
Mean (SD)	72.6 ± 20.6	73.7 ± 19.5
Median (25th–75th percentile)	69.4 (58.5–82.5)	70.5 (60.7–83.6)
HbA1c (%)^a^		
Mean (SD)	8.8 ± 4.0	8.9 ± 3.9
Median (25th–75th percentile)	8.5 (7.5–9.7)	8.6 (7.7–9.8)

Data presented as mean ± SD or median (25th–75th percentile)

^a^Number of measurements in study period: 41,583 for all participants group, 16,989 for participants contributing for 10 years and above

**Fig. 1:**
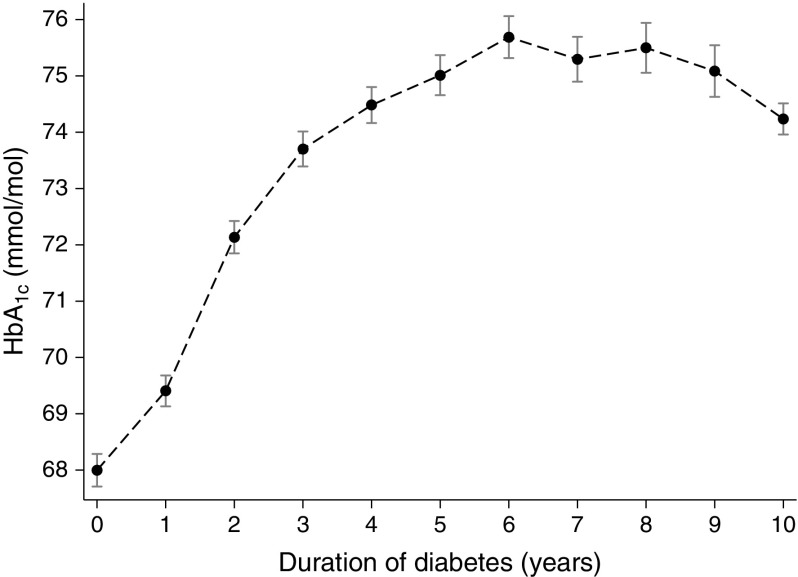
Mean HbA_1c_ by year from diagnosis of type 1 diabetes in 4525 patients with newly diagnosed diabetes. For example, duration time 0 represents the HbA_1c_ values captured from date of diagnosis to year 1; time 1 represents the HbA_1c_ measurements from year 1 to 2, and time 9 represents HbA1c measurements from year 9 to 10

HbA_1c_ increased with time from diagnosis in individuals with type 1 diabetes and stabilised by 5 years to an average of 75.0 mmol/mol (9.0%) following this period (Fig. 1). However, the timing of stabilisation was dependent on age at diagnosis and sex (ESM Fig. 1).

**Fig. 2:**
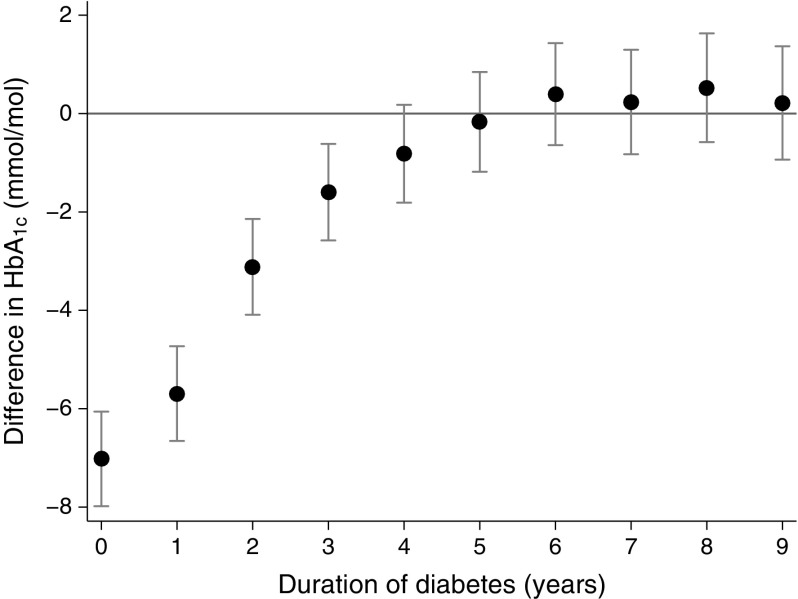
Mean (95% CI) difference in HbA_1c_ between the mean for the reference group (≥10 years post diagnosis) and the mean for each year after diagnosis. Duration time 0 represents the HbA_1c_ values captured from date of diagnosis to year 1; time 1 represents the HbA_1c_ measurements from year 1 to 2, etc. Models were constructed using a random intercept and slopes model adjusting for age, sex and Townsend index. The time point at which the 95% CI for the yearly difference crosses the null value (zero) is considered the starting point of tracking

The largest difference between mean HbA_1c_ measurements was between those taken in the first year following diagnosis (time: 0 years in Fig. 2) and those taken after 10 or more years (the reference category) (−7.0 [95% CI −8.0, −6.1] mmol/mol [0.6%]; Fig. 2). The mean HbA_1c_ difference for the successive periods after diagnosis (1–2 years, 2–3 years, etc.) compared with after 10 or more years declined considerably and was no longer statistically significant 5 years following diagnosis (a duration time of 4 years in figures illustrates HbA_1c_s measured between 4 and 5 years after diagnosis); the mean HbA_1c_ difference for this duration after diagnosis was −0.8 (95% CI −1.8, 0.2) mmol/mol (0.7%). The findings remained similar when analysis was restricted only to participants contributing HbA_1c_ measurements for 10 years and above (ESM Fig. 2).

**Fig. 3:**
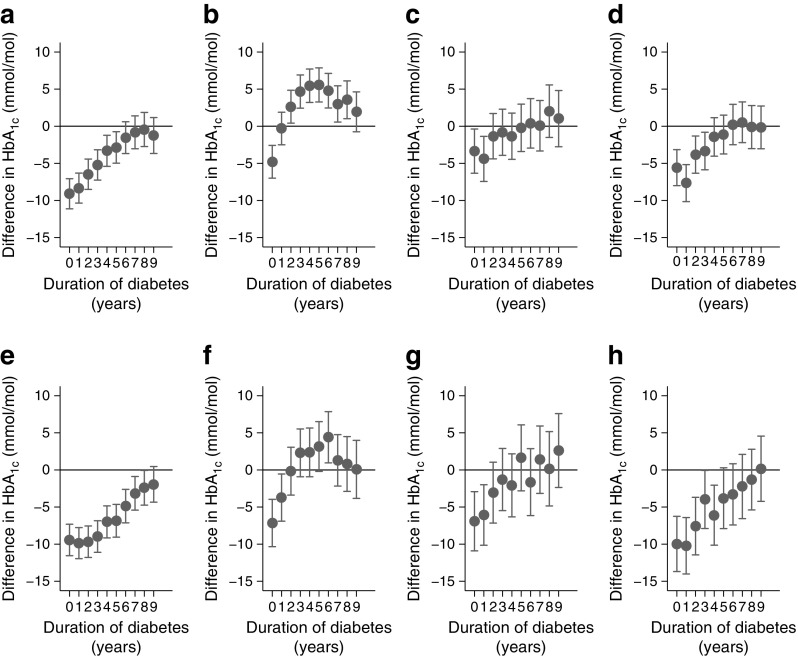
Difference in HbA_1c_ between the reference group (≥10 years) and each year after the time of diagnosis of type 1 diabetes stratified by age at diagnosis and sex. Duration time 0 represents the HbA_1c_ values captured from date of diagnosis to year 1; time 1 represents the HbA_1c_ measurements from year 1 to 2, etc. (**a**, **e**) Diagnosis between ages 0 and 10 years; (**b**, **f**) between ages 10 and 20 years; (**c**, **g**) between ages 20 and 30 years; (**d**, **h**) between ages 30 and 40 years; male group (**a**–**d**) and female group (**e**–**h**). Models were constructed using a random intercept and slopes model adjusting for age, sex and Townsend index. The difference is given for each year with a point estimate and its 95% CI. The time point where the above difference crosses the null value (zero) is considered the starting point of tracking

There was considerable heterogeneity in the timing of tracking in the stratified analysis using sex and age group. Adult onset type 1 diabetes seem to track earlier (Fig. 3) and at a lower mean HbA_1c_ (ESM Fig. 1). Children diagnosed between 0 and 10 years tracked from 6–7 years and 8–9 years after diagnosis for boys and girls, respectively (Fig. 3a, e). For the 10–20 years diagnosis age group, tracking occurred at 9–10 years in males and 7–8 years in females (Fig. 3b, f). For the 20–30 years group, tracking occurred at 2–3 years for both sexes (Fig. 3c, g). Among the 30–40 years age group, tracking was at 4–5 years in men (Fig. 3d) and 5–6 years in women (Fig. 3h).

## Discussion

We show that in individuals with type 1 diabetes, glycaemic control measured by HbA_1c_ settles onto a long-term ‘track’ and that this occurs on average by 5 years following diagnosis. We also show that the age at diagnosis modifies both the rate at which individuals settle into their track and the absolute HbA_1c_ tracking level for the next 10 years. Our analytical approach utilising a random intercept and slope model ensures that our overall results are not confounded by trajectories at an individual level.

This is the first study to demonstrate the phenomenon of glycaemic tracking on a large number of unselected, newly diagnosed individuals with type 1 diabetes across a broad age group and with a long period of follow-up. Crucially, this study is unique in that the association has been adjusted for important potential confounders such as local clinical practice and Townsend score. The study is weakened by the fact that adequate follow-up data are not available for greater than 10 years and that the diagnosis of type 1 diabetes was taken prima facie from the national THIN general practice database. For this reason, we limited the analysis to those diagnosed with type 1 diabetes under the age of 40 years, on insulin alone and with no history of oral hypoglycaemic prescriptions. Finally, the HbA_1c_ assay has evolved over the course of the time span during which this study extends. Although appropriate conversions were meticulously undertaken, inherent differences in methodologies should be taken into account when interpreting differences in HbA_1c_ before and after 2009 in the UK. Similarly, several important changes in diabetes management have been introduced over the study period such as the introduction of novel insulin formulations, widespread adoption of intensive insulin treatment schemes and revision of education systems. These may have contributed towards an improvement of glycaemic control at a national level and although data is missing, they should be considered in the context of this study.

It is notable that the overall HbA_1c_ levels in the UK are suboptimal. This has been seen before with regard to other countries [28]. Furthermore there is a discrepancy between the sexes and this may relate to the higher insulin resistance [29] and behavioural issues [30] that may be more prevalent in younger women and girls with type 1 diabetes.

Several hypotheses, focusing on endogenous and/or exogenous factors, have been suggested to underlie the phenomenon of glycaemic tracking. Residual beta cell function, measured by stimulated C-peptide, decays with time following type 1 diabetes diagnosis [31] and low C-peptide is associated with higher HbA_1c_ [32, 33]. Therefore, the loss of C-peptide following diagnosis may explain the rising HbA_1c_ in the initial 5 years. Alternatively, or in addition, individual patient-related factors may contribute. Habituation of the day to day approach to managing chronic disease can make any long-lasting change difficult [34]. Studies involving paediatric and adolescent individuals with type 1 diabetes indicate that age, sex, body mass index, socioeconomic factors, physical activity levels, frequency of glucose monitoring and personality traits can also influence temporal HbA_1c_ trends [16, 35–37]. It may be that a combination of these endogenous and exogenous factors contributes to the phenomenon of tracking and that both need to be addressed for effective long-term glycaemic control.

There are two clear implications of our results. First, there is a 5 year window during which longer-term HbA_1c_ and therefore risk of diabetes complications is determined. Thus, urgent and appropriate targeting of therapies to this period of type 1 diabetes natural history should be considered. This may include, for example, the development of a newly diagnosed type 1 diabetes pathway with structured education and aggressive glucose control. Second, the benefits of addressing HbA_1c_ after the first 5 years should be explored. It has been suggested that efforts would be better directed at risk factors other than HbA_1c_ in individuals with established type 1 diabetes [13]. However, such an approach may result in loss of the tracking phenomenon and deterioration in HbA_1c_ so this needs to be carefully explored. Unfortunately, data are missing with regards to both effectiveness and timing of a focused clinical intervention targeted at changing the track and this clinical need becomes even more evident in light of the study findings. Furthermore, it is worth noting that HbA_1c_ provides only one indication of glycaemic control. Day to day glucose variability is of greater concern to individuals and is poorly reflected in the HbA_1c_ measure. Indeed, ‘real world’ experience of new interventions such as structured education and insulin pump therapy suggest they have had a greater benefit on glucose variability than meaningful long-term changes to HbA_1c_ [38, 39].

This is the first comprehensive study of the phenomenon of HbA_1c_ tracking and supports the need to optimise glycaemic control early in the natural history of type 1 diabetes. Studies are now needed to explore the mechanisms underlying this phenomenon and how best to optimise tracking in newly diagnosed individuals.

## Electronic supplementary material

ESM (PDF 243 kb)

## Data Availability

THIN data governance does not allow us to share individual patient data and therefore where possible metadata is presented. Researchers may apply for individual patient data access at https://www.iqvia.com/contact. The authors declare that there is no duality of interest associated with this manuscript.

## Abbreviation

THIN: The Health Improvement Network
