# Supplementary material for: Clinically meaningful and lasting HbA1c improvement rarely occurs after 5 years of type 1 diabetes: an argument for early, targeted and aggressive intervention following diagnosis
Source: Diabetologia. 2018 Feb 24;61(5):1064–70. doi: 10.1007/s00125-018-4574-6 (PMC6448997; doi:10.1007/s00125-018-4574-6)
Supplement: Supplementary file 1 — (PDF 243 kb) [file 125_2018_4574_MOESM1_ESM.pdf]

ESM Table 1

Study	Year	Number of patients	Age at study entry *	Duration of follow-up (years)	Other findings	Limitations
Forsander [1]	1998	38	3-15 years	5	Patients with poor HbA1c at five years of diabetes can be identified by the second year	Prevalent cases, small cohort size, paediatric population, short duration of follow up
Jorde [2]	2000	214	30.4 ± 11.9	6	The coefficient of variation for HbA1c was negatively related to age, and accordingly, to years on insulin	Small cohort size, short duration of follow up, different statistical approach
Luyckx [3]	2009	72	13	10	Family climate and self-concept associate with higher HbA1c	Prevalent cases, small cohort size, paediatric population
Edge [4]	2010	362	0–18 years	15	HbA1c levels at 6 months after diagnosis increased with age with a quadratic fit	Prevalent cases, small cohort size, different statistical approach, no information on gender
Helgeson [5]	2010	132	12	5	Low social status, BMI, pubertal status associate with higher subsequent HbA1c	Prevalent cases, small cohort size, paediatric population
Viswanathan [6]	2011	120	7.6 ± 3.9	4	No other baseline variables correlated with subsequent glycaemic control.	Prevalent cases, short duration of follow up, small cohort size, paediatric population
Jackson [7]	2012	155	6.6	14	HbA1c levels at diagnosis and within the first year were significant predictors of long-term control	Prevalent cases, small cohort size, paediatric population
Shalitin [8]	2012	173	3.8 ± 1.6	7	The findings was not dependent on the type of insulin regimens	Prevalent cases, paediatric population, small cohort size
Cabrera [9]	2013	138	6.8 ± 3.3	5	Education and location at time of diagnosis do not appear to play a	Prevalent cases, short duration of follow up,

					significant role in long-term glycaemic control.	paediatric population, no direct exploration of tracking, small cohort size, different primary objective
Gill [10]	2013	181	41 ± 8	5	Small improvements can occur in specific sub-groups – notably males and those with poor baseline control.	Prevalent cases, small cohort size, short duration of follow up
Samuelsson [11]	2013	1,543	5-19 years	12	Girls have higher mean HbA1c than boys.	Prevalent cases, different statistical approach, paediatric population
Clements [12]	2014	2218	0-20	5	Teenage years and black ethnicity associate with higher HbA1c	Short duration of follow up, paediatric population
Hofer [13]	2014	1,146	7.2 (IQR 4.7-9.4)	20	A significant increase in HbA1c is seen during puberty	Different statistical approach
Lawes [14]	2014	155	7.9 (4.5 - 10.9)	5	The relationship between HbA1c at 6 months after diagnosis and future metabolic control was independent of potential patient and observation level confounders.	Prevalent cases, small cohort size, short duration of follow up, paediatric population,
Clements [15]	2016	8,774	8-18 and 16-26 years		Elevated HbA1c in 16 – 18 yr-olds begin a steady improvement into early adulthood. Gender failed to show a clinically significant association with mean age-centered HbA1c	Prevalent cases
Schwandt [16]	2017	6,433	8 years	11	Distinct HbA1c trajectories over the period of follow up can be predicted by BMI, frequency of glucose testing and physical activity levels	Prevalent cases, narrow age range included

ESM Table 1. Previous studies exploring the phenomenon of glycaemic tracking listed in chronological order.
Presented as mean ± standard deviation, median (interquartile range) or minimum-maximum, HbA1c: Glycated Haemoglobin,

References

[1] Forsander G, Persson B, Sundelin J, Berglund E, Snellman K, Hellstrom R (1998) Metabolic control in children with insulin-dependent diabetes mellitus 5 y after diagnosis. Early detection of patients at risk for poor metabolic control. Acta Paediatr 87: 857-864

[2] Jorde R, Sundsfjord J (2000) Intra-individual variability and longitudinal changes in glycaemic control in patients with Type 1 diabetes mellitus. Diabet Med 17: 451-456

[3] Luyckx K, Seiffge-Krenke I (2009) Continuity and change in glycemic control trajectories from adolescence to emerging adulthood: relationships with family climate and self-concept in type 1 diabetes. Diabetes Care 32: 797-801

[4] Edge JA, James T, Shine B (2010) Persistent individual tracking within overall improvement in HbA1c in a UK paediatric diabetes clinic over 15 years. Diabet Med 27: 1284-1288

[5] Helgeson VS, Snyder PR, Seltman H, Escobar O, Becker D, Siminerio L (2010) Brief report: trajectories of glycemic control over early to middle adolescence. J Pediatr Psychol 35: 1161-1167

[6] Viswanathan V, Sneeringer MR, Miller A, Eugster EA, DiMeglio LA (2011) The utility of hemoglobin A1c at diagnosis for prediction of future glycemic control in children with type 1 diabetes. Diabetes Res Clin Pract 92: 65-68

[7] Jackson C, Wernham, EM, Elder, CJ , Wright, NP (2013) Early glycaemic control is predictive of long-termcontrol: a retrospective observational study. Practical Diabetes 30: 16-18

[8] Shalitin S, Phillip M (2012) Which factors predict glycemic control in children diagnosed with type 1 diabetes before 6.5 years of age? Acta Diabetol 49: 355-362

[9] Cabrera SM, Srivastava NT, Behzadi JM, Pottorff TM, Dimeglio LA, Walvoord EC (2013) Long-term glycemic control as a result of initial education for children with new onset type 1 diabetes: does the setting matter? Diabetes Educ 39: 187-194

[10] GV Gill WM, Wallymahmed A, MacFarlane IA, Woodward A (2013) Glycaemic streaming in type 1 diabetes: implications for intervention? . Practical Diabetes 30: 229–232

[11] Samuelsson U, Steineck I, Gubbjornsdottir S (2014) A high mean-HbA1c value 3-15 months after diagnosis of type 1 diabetes in childhood is related to metabolic control, macroalbuminuria, and retinopathy in early adulthood--a pilot study using two nation-wide population based quality registries. Pediatr Diabetes 15: 229-235

[12] Clements MA, Lind M, Raman S, et al. (2014) Age at diagnosis predicts deterioration in glycaemic control among children and adolescents with type 1 diabetes. BMJ Open Diabetes Res Care 2: e000039

[13] Hofer SE, Raile K, Frohlich-Reiterer E, et al. (2014) Tracking of metabolic control from childhood to young adulthood in type 1 diabetes. J Pediatr 165: 956-961 e951-952

[14] Lawes T, Franklin V, Farmer G (2014) HbA1c tracking and bio-psychosocial determinants of glycaemic control in children and adolescents with type 1 diabetes: retrospective cohort study and multilevel analysis. Pediatr Diabetes 15: 372-383

[15] Clements MA, Foster NC, Maahs DM, et al. (2016) Hemoglobin A1c (HbA1c) changes over time among adolescent and young adult participants in the T1D exchange clinic registry. Pediatr Diabetes 17: 327-336

[16] Schwandt A, Hermann JM, Rosenbauer J, et al. (2017) Longitudinal Trajectories of Metabolic Control From Childhood to Young Adulthood in Type 1 Diabetes From a Large German/Austrian Registry: A Group-Based Modeling Approach. Diabetes Care 40: 309-316

ESM Figure 1

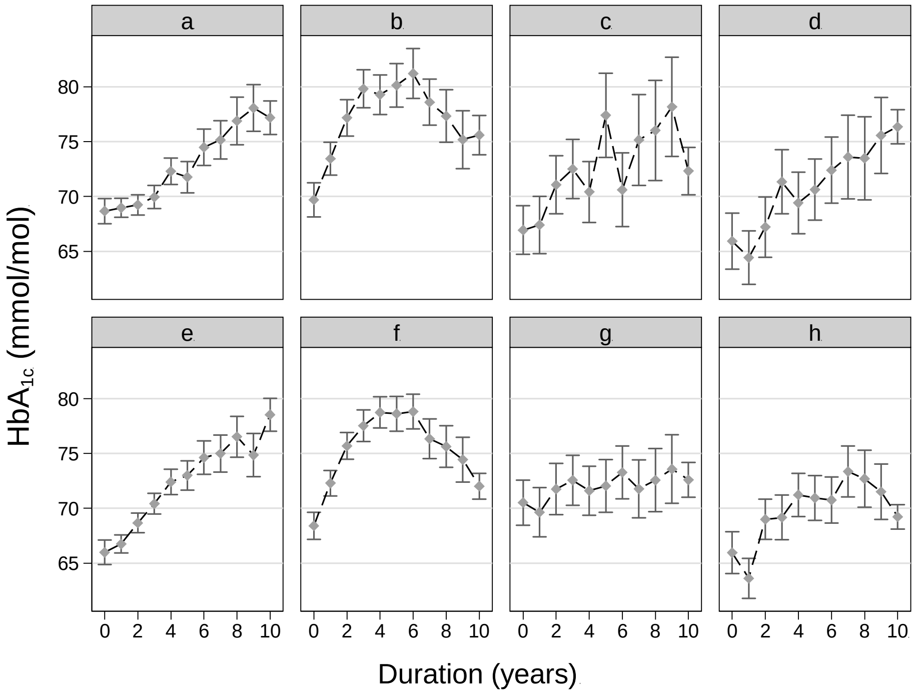

ESM Fig 1: HbA_{1c} change with time from diagnosis in individuals with type 1 diabetes displayed according to their sex and age range at which they were diagnosed. Males are panels (a-d). Females are panels (e-h). Age group 0-10 years are panels (a,e), age group 10-20 are panels (b,f), age group 20-30 are panels (c,g), age groups 30-40 are panels (d,h). Duration time 0 represents the HbA1c values captured from date of diagnosis to year 1; time 1 represents the HbA_{1c} measurements from year 1 to 2, etc.

ESM Figure 2

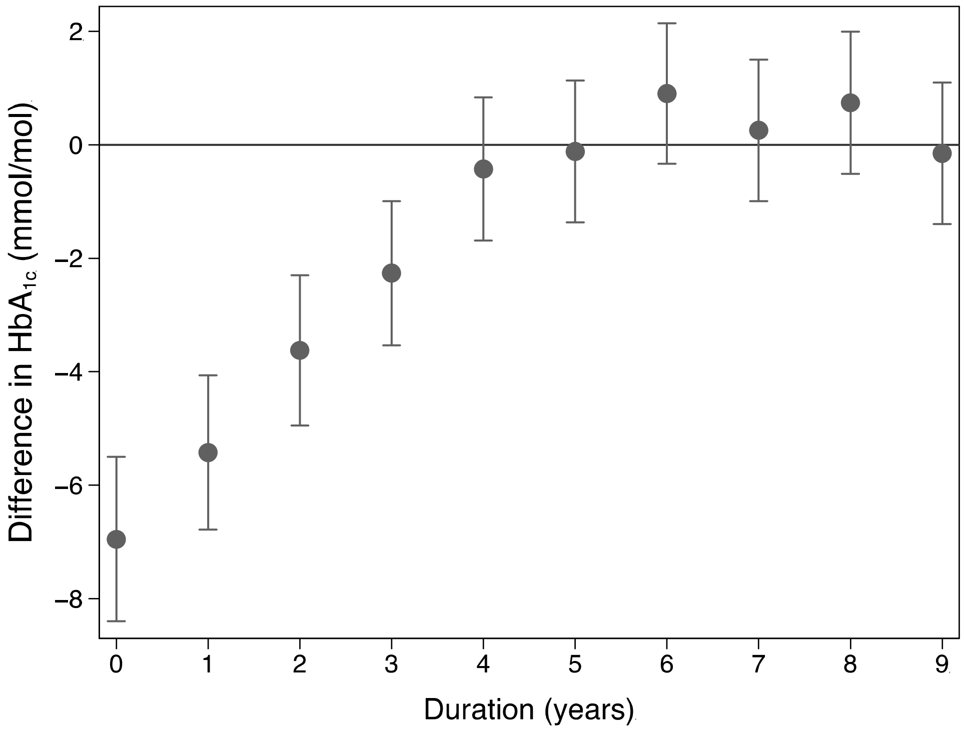

ESM Fig 2: HbA_{1c} change with time from diagnosis in individuals with type 1 diabetes. Random intercept and slopes model-Adjusted for sex, age and Townsend index. (restricted to individuals with 10 years or more of follow-up). Duration time 0 represents the HbA1c values captured from date of diagnosis to year 1; time 1 represents the HbA_{1c} measurements from year 1 to 2, etc.
